# Low expression of acyl-CoA thioesterase 13 is associated with poor prognosis in ovarian serous cystadenocarcinoma

**DOI:** 10.3389/fgene.2023.1213022

**Published:** 2023-06-22

**Authors:** Xiaofeng Lv, Weijiao Wang, Xiaoyu Liu, Yuhuan Liu, Lili Guo, Changyu Wang

**Affiliations:** ^1^Department of Obstetrics and Gynecology, Tongji Hospital, Tongji Medical College, Huazhong University of Science and Technology, Wuhan, China; ^2^ Cancer Biology Research Center, Tongji Hospital, Tongji Medical College, Huazhong University of Science and Technology, Wuhan, China

**Keywords:** ACOT13, prognosis, TCGA, ovarian serous cystadenocarcinoma, tumor staging, immune checkpoint

## Abstract

**Objective:** Acyl-CoA thioesterase 13 (ACOT13) encodes a member of the thioesterase superfamily. It has not been reported in ovarian cancer. This research aimed at evaluating the expression and prognostic value of ACOT13 in ovarian serous cystadenocarcinoma (OSC).

**Methods:** We extracted and analyzed TCGA, GEPIA, THPA, GTEx, miRWalk, and GDSC databases to investigate the potential carcinogenic mechanism of ACOT13 in OSC, including the correlation of ACOT13 with prognosis, immune checkpoint, tumor mutational burden (TMB), and 50% inhibition concentration (IC50) score. The incidence of endpoint events was compared with Kaplan-Meier survival analysis. Independent prognostic factors for OSC were evaluated with univariate and multivariate Cox regression analyses, and a nomogram was established.

**Results:** The expression of ACOT13 was increased in OSC and correlated with tumor stage, with higher expression in stages I and II than in stages III and IV. Besides, it was observed that low expression of ACOT13 is correlated with poor overall survival (OS), progression-free survival (PFS), and disease-specific survival (DSS) in patients with OSC. There was a positive correlation between ACOT13 expression and immune checkpoint sialic acid-binding Ig-like lectin (SIGLEC) 15 and TMB. Patients with low ACOT13 expression had higher cisplatin IC50 scores.

**Conclusion:** ACOT13 is an independent prognostic factor and a promising clinical target for OSC. In the future, the carcinogenic mechanism and clinical application value of ACOT13 in ovarian cancer need to be further studied.

## 1 Introduction

ACOT13 can hydrolyze fatty acyl-CoA to form free fatty acid (FFA) and coenzyme A (CoA), which exert a significant effect on metabolizing energy (Tillander et al., 2017). The structure of ACOT13 is a so-called “HotDog” domain (Cantu et al., 2014). It is described as a seven-strand antiparallel beta-folded “bread” wrapped around a hydrophobic curved alpha-spiral “sausage”, as well as containing layers made up of loops on the helix as a cover ring (Leesong et al., 1996). According to past research, ACOT13 is related to the development of lung cancer (Liu et al., 2018) and autosomal dominant polycystic kidney disease (Du et al., 2021). Interestingly, the effect of ACOT13 on gynecological tumors has not been reported.

Ovarian cancer acts as the most lethal malignancy in gynecology. Because of non-specific, insidious clinical manifestations and lack of sensitive tumor markers, approximately 70% of patients with ovarian cancer are diagnosed with advanced stage (FIGO stage III or stage IV) (Stewart et al., 2019). Ovarian serous cystadenocarcinoma is the most ordinary histological subtype of ovarian cancer, occupying 75%–80% of all ovarian cancers (Escalona et al., 2018). First-line treatment of ovarian cancer includes surgery and chemotherapy. Although chemotherapy and targeted therapy have greatly increased the short-term efficacy of ovarian cancer patients, they still cannot solve the problems of drug resistance and recurrence of ovarian cancer, and the overall survival of patients has not been significantly improved (Siegel et al., 2023). Therefore, new prognostic factors for ovarian cancer need to be identified.

This research fully explored the expression of ACOT13 in OSC and its association with prognosis, immune checkpoint, TMB, and chemotherapeutic agents. In addition, the prognostic value of ACOT13 in pan-carcinoma and the miRNA that might regulate its expression were further investigated. This study aimed at evaluating whether ACOT13 is a prognostic marker for OSC.

## 2 Materials and methods

### 2.1 Differential expression analysis

We obtained RNAseq data (level 3) and corresponding clinical information for 376 OSC patients from the Cancer Genome Atlas (TCGA) dataset (https://portal.gdc.cancer.gov/) (Zhou et al., 2020). Firstly, click the link to enter the page and select the “Repository” button, then click the “Case” option to select “TCGA” and tumor type, and then select the corresponding clinical information for download in the “Files” interface. GTEx database (https://gtexportal.org/home/datasets) provided 180 cases of normal ovary RNA sequencing data (GTEx Consortium, 2020). The R software “ggplot2” package was adopted for expression difference analysis. Wilcox test was used to compare mRNA expression differences between normal and tumor tissues. False discovery rate (FDR) of 0.05 was used as the cutoff value. To further demonstrate the difference in the expression of ACOT13 in ovarian cancer and normal tissues, boxplot was plotted in the GEPIA database (http://gepia.cancer-pku.cn/index.html) (Tang et al., 2017). Enter “ACOT13” for gene name, “OV” for tumor type, and default values for other Settings.

### 2.2 Immunohistochemistry

The immunohistochemical results of ACOT13 in normal ovarian tissue and ovarian cancer tissue were obtained from THPA database (https://www.proteinatlas.org/) (Uhlen et al., 2017) to compare the difference in protein level expression. After entering the database, “ACOT13” was input for retrieval, and “TISSUE” and “PATHOLOGY” were selected to examine the protein expression in tissues. The antibody numbers were HPA019881 and HPA057134, respectively.

### 2.3 Identification of prognostic factors in OSC

The prognostic effects of ACOT13, age, race, tumor stage, and tumor grade were evaluated with univariate and multivariate Cox regression analysis. The “forestplot” package was used to build the forest. On basis of the outcomes of the multivariate Cox proportional risk analysis, a nomogram was developed to forecast the recurrence rate at 1, 2, and 3 years with the “rms” package.

### 2.4 Relationship between ACOT13 and the survival of OSC

By extracting survival information from each sample in the TCGA database, we used OS, PFS, DSS, and disease-free survival (DFS) to evaluate the association between ACOT13 expression and prognosis in patients with ovarian cancer. According to the survival information of each patient, Kaplan-Meier curves were plotted applying the “survival” and “survminer” packages of R software. For Kaplan-Meier curves, *p*-values and hazard ratio (HR) with 95% confidence interval (CI) were generated by log-rank tests and univariate cox proportional hazards regression. HR represents the risk coefficient of the high expression group relative to the low expression group. If HR is greater than 1, it means that the gene is a risk factor; if HR is less than 1, it means that the gene is a protective factor. 95% CI represents HR confidence interval.

### 2.5 Association of ACOT13 with immune checkpoint, TMB and drug IC50

The TCGA dataset was used to download RNA-sequencing expression profiles for OSC and their relevant clinical data. The expression values of these 8 common immune checkpoint genes were extracted, and the correlation analysis of the immune checkpoints was performed using R software “ggplot2” package. Use the “ggstatsplot” package for TMB correlation analysis. The correlation between ACOT13 and the infiltration level of 6 types of immune cells was studied using TIMER (https://cistrome.shinyapps.io/timer/) database (Li et al., 2017). The drug sensitivity score of each sample was predicted from the GDSC database (Iorio et al., 2016). The forecast process was carried out by the R software “pRRophetic” package, in which the maximum IC50 of the sample was estimated by ridge regression, all parameters were set as defaults, batch effects of combat and tissue types of ALL were used, and duplicate gene expression was sum up as average value.

### 2.6 Expression and prognosis of ACOT13 in pan-carcinoma

The TCGA and GTEx databases were used to obtain RNAseq and clinical data of 33 tumors. The “forestplot” R package was adopted to make univariate Cox regression analysis and forest plots. Differences in ACOT13 expression were detected by rank sum test.

### 2.7 Prediction of miRNA that regulate ACOT13 expression

First, the target miRNA of ACOT13 was downloaded from the miRWalk (http://mirwalk.umm.uni-heidelberg.de/) database to remove duplicates. Then, combined with TCGA database RNAseq data, R software “pheatmap” package is used for correlation analysis.

### 2.8 Statistical analysis

SPSS (V23.0 IBM Corp., Armonk, NY, United States) statistical software and R software (R3.6, R Core Team, Vienna, Austria) were adopted for data analysis. Measurements are shown as mean ± SD. The Wilcox test was adopted to compare the statistical difference of two groups, and Kruskal-Wallis test was adopted for testing the significance difference of three groups. Diversities in survival between these groups were compared with Log-rank test. Spearman’s correlation analysis to illustrate the correlation between quantitative variables without a normal distribution. p < 0.05 was considered statistically significant.

## 3 Results

### 3.1 Comparison of clinical features in patients with OSC

The flow diagram of this study was shown in Supplementary Figure S1. Patients were fallen into two groups on basis of ACOT13 expression levels, and Table 1 showed the clinical features. There were no significant differences in age, race, tumor grade, or recurrence between the two groups. It was observed, however, that patients with low expression and high expression had significantly different tumor stages (*p* < 0.05). Sankey chart of patient clinical information was shown in Supplementary Figure S2.

**TABLE 1:** Clinical characteristics of patients with high and low expression of ACOT13.

Characteristics		High ACOT13 express	Low ACOT13 express	*p*-value
Age	Mean (SD)	59.1 (10.9)	60.1 (11.7)	
	Median [MIN, MAX]	58 [30,87]	59 [34,87]	0.354
Race	AMERICAN INDIAN	1	1	
	ASIAN	8	3	
	BLACK	15	10	
	WHITE	161	165	
	ISLANDER		1	0.358
pTNM stage	IC	1		
	IIA	2	1	
	IIB	13	5	
	IIIA	4	3	
	IIIB	10	4	
	IIIC	139	133	
	IV	18	40	0.008
Grade	G1		1	
	G2	31	19	
	G3	157	166	0.112
New tumor event type	Progression	6	12	
	Recurrence	105	94	0.183
Radiation therapy	Non-radiation	5	1	
History of neoadjuvant treatment	No neoadjuvant	188	187	
	Neoadjuvant		1	

### 3.2 ACOT13 expression differs between OSC and normal tissues

**FIGURE 1:**
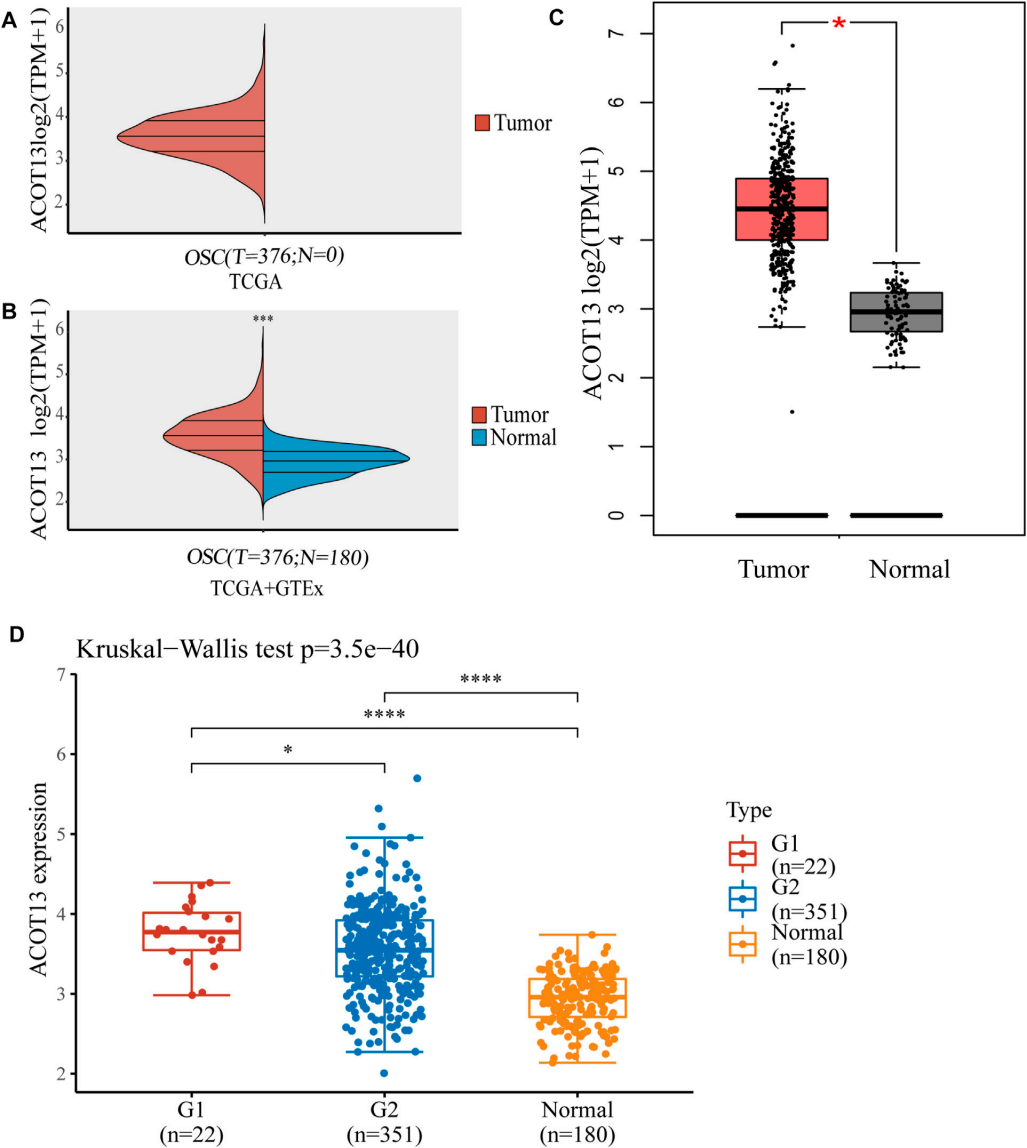
Expression level of ACOT13 mRNA in different stages and normal tissues. **(A,B)** Expression levels of ACOT13 in OSC and normal tissues. **(C)** Differences in ACOT13 expression between tumor and normal tissues in GEPIA database. **(D)** ACOT13 expression levels in patients with different tumor stages. *p < 0.05, ***p < 0.001, ****p < 0.0001.

**FIGURE 2:**
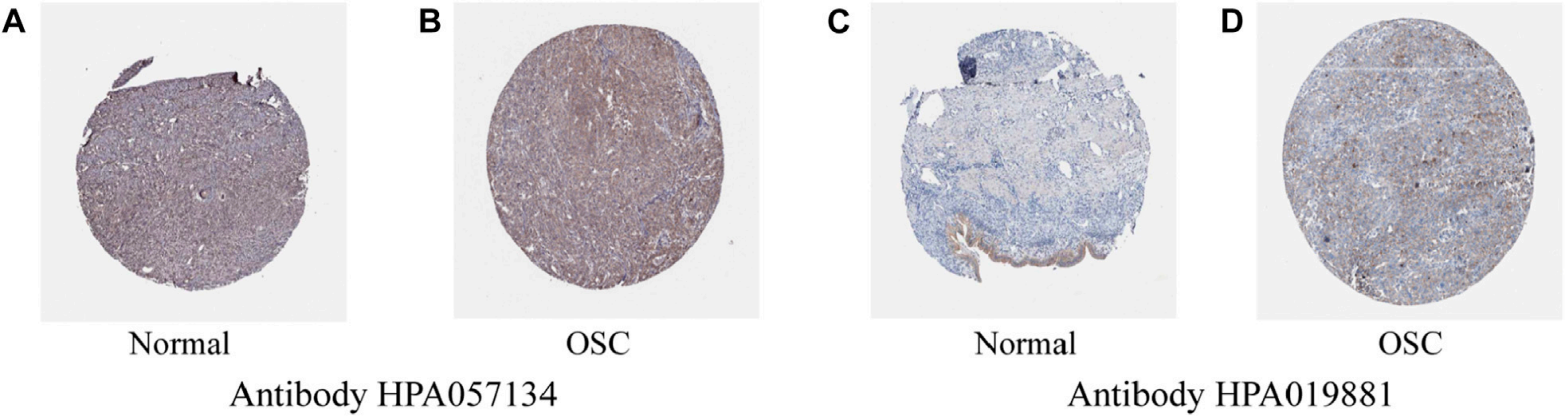
**(A,C)** Expression of ACOT13 in normal ovarian tissue. **(B,D)** Expression of ACOT13 in OSC.

To assess differences in ACOT13 expression, RNAseq data from 376 OSC patients in TCGA were analyzed using R software. On account of the lack of normal ovarian tissue information in the TCGA database, 180 normal samples from the GTEx database were used as controls. It was found that ACOT13 expression was elevated in OSC (Figures 1A, B). We further verified the expression of ACOT13 in GEPIA database (Figure 1C). Besides, the expression of ACOT13 was correlated with tumor stage, and the expression level of ACOT13 was higher in stages I-II (G1) than in stages III-IV (G2) (Figure 1D). From the THPA database, immunohistochemical images were analyzed to determine whether ACOT13 is differentially expressed in OSC protein levels. We found that ACOT13 protein was mainly localized in the cytoplasm, with low expression in normal ovarian tissue and medium to high intensity staining in tumor tissue (Figure 2).

### 3.3 Prognostic value of ACOT13 in patients with OSC

**FIGURE 3:**
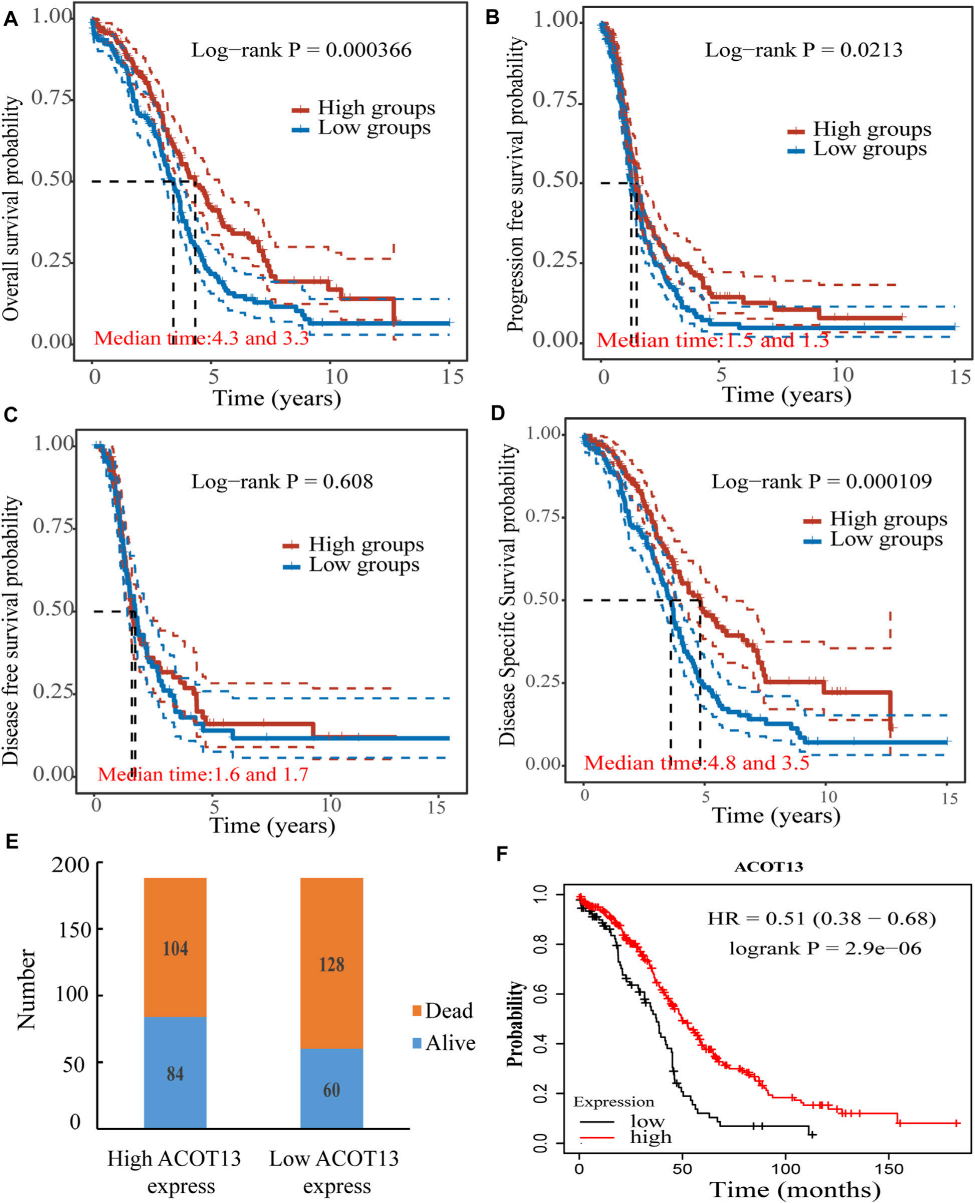
Correlation between ACOT13 expression and OS **(A)**, PFS **(B)**, DFS **(C)** and DSS **(D)**. **(E)** Stacked bar charts show the proportion of patients in both groups who survived or died. **(F)** Kaplan-Meier Plotter database verified the correlation between ACOT13 and OS.

To examine the association between ACOT13 and prognosis in patients with OSC, we analyzed OS, PFS, disease-free survival (DFS), and DSS in patients with high or low ACOT13 expression. It was observed that the OS (*p* < 0.001), PFS (*p* < 0.05) and DSS (*p* < 0.001) of patients in the low expression group of ACOT13 were significantly lower than those in the high expression group (Figures 3A–D). The stacked bar diagram was shown in Figure 3E. A separate ovarian cancer cohort from the Kaplan-Meier Plotter database (http://kmplot.com/analysis/index.php?p=background) confirmed similar results (Figure 3F, *p* < 0.0001).

**FIGURE 4:**
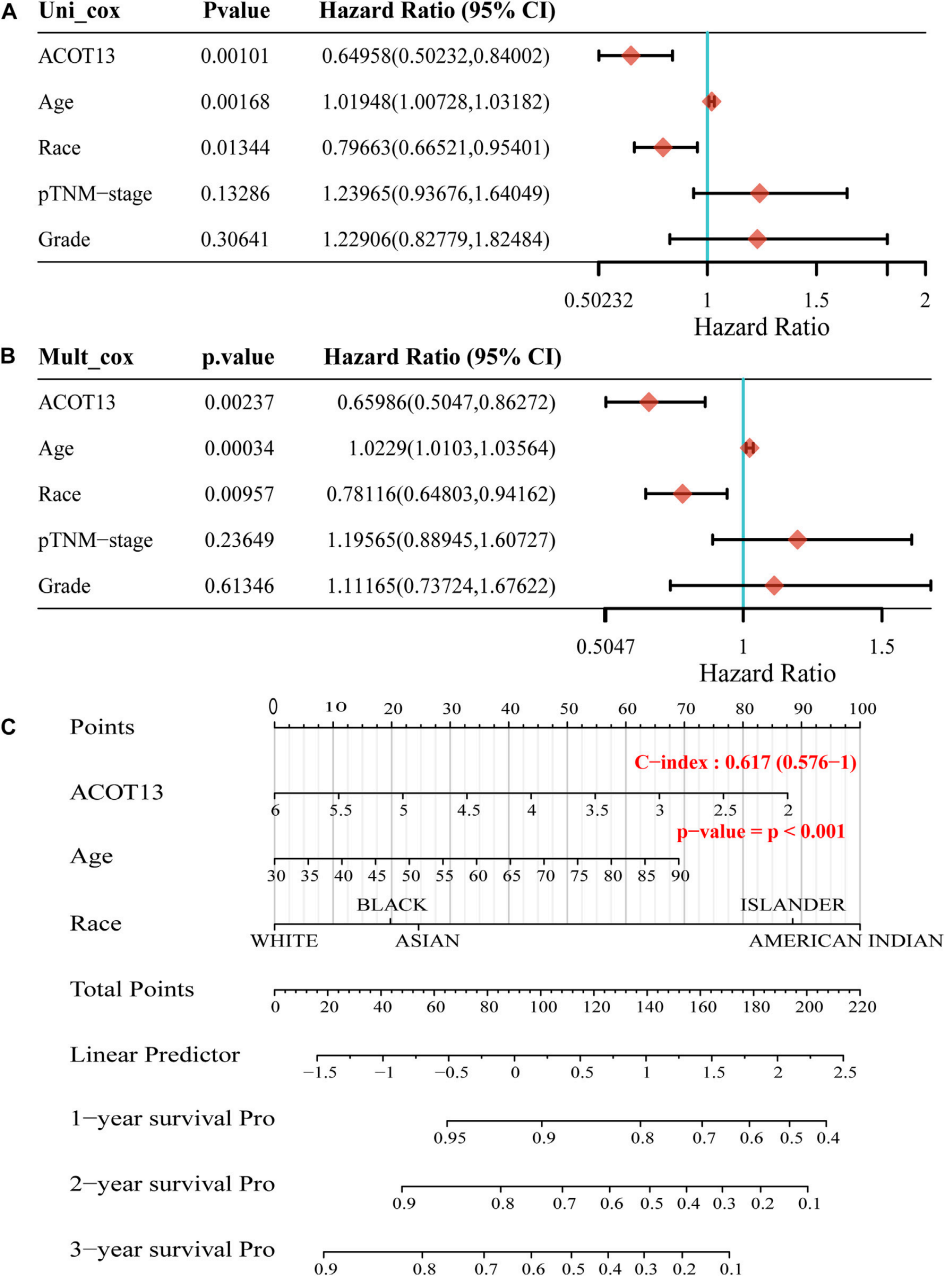
The *p*-value, hazard ratio, and confidence interval are explored by univariate **(A)** and multivariate **(B)** Cox regression. **(C)** The nomogram predicts 1, 2–and 3-year overall survival of OSC patients.

To further verify the prognostic value of ACOT13 in OSC, univariate and multivariate Cox regression analyses were made based on the clinical characteristics of patients. According to univariate Cox regression analysis, ACOT13 (HR = 0.64958, *p* < 0.01), age (HR = 1.01948, *p* < 0.01) and race (HR = 0.79663, *p* < 0.05) had predictive value for OS in OSC patients (Figure 4A). Additionally, in subsequent multivariate cox regression analysis, ACOT13 (HR = 0.65986, *p* < 0.01), age (HR = 1.0229, *p* < 0.001) and race (HR = 0.78116, *p* < 0.01) continued to have predictive value for OS in OSC patients (Figure 4B). A nomogram was constructed based on variables significantly associated with OSC outcomes to graphically display 1-year, 2-year, and 3-year overall survival (Figure 4C). Analysis of pan-cancer data showed that ACOT13 also has disease prognostic potential in kidney chromophobe (KICH) and kidney renal clear cell carcinoma (KIRC). Violin plot and forest plot are shown in Supplementary Figures S3, S4, respectively.

### 3.4 Association of ACOT13 with immune checkpoint, TMB and drug IC50

**FIGURE 5:**
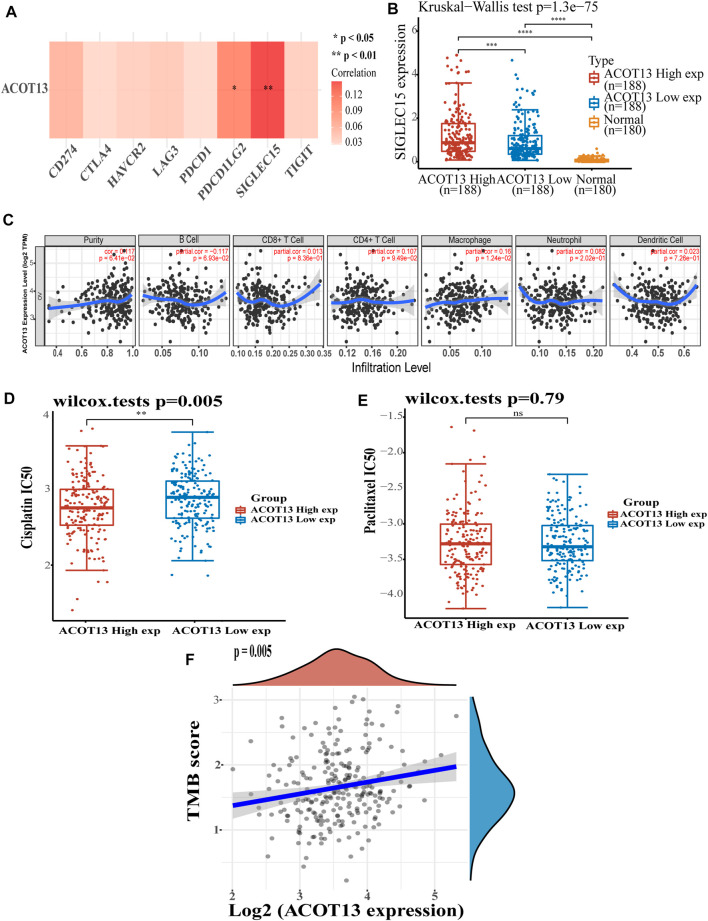
**(A)** ACOT13 and immune checkpoint expression heat map. **(B)** SIGLEC15 was expressed at higher levels in patients with OSC who had high ACOT13 expression. **(C)** Association between ACOT13 expression and immune cell infiltration. **(D,E)** Correlation between ACOT13 expression and IC50 scores of cisplatin and paclitaxel. **(F)** Correlation analysis of ACOT13 expression and TMB in OSC patients.

We further explored the potential mechanism by which low expression of ACOT13 results in poor prognosis in OSC. It was observed that ACOT13 was significantly positively associated with immune checkpoint PDCD1LG2 (*p* < 0.05) and SIGLEC15 (*p* < 0.01) (Figure 5A). In patients with high levels of ACOT13 expression, SIGLEC15 expression was higher than in patients with low levels of ACOT13 expression (Figure 5B, *p* < 0.001). Immunocell infiltration analysis showed a significant positive correlation between ACOT13 and macrophages (Figure 5C, *p* < 0.05). This suggested that ACOT13 exerts a great effect on tumor formation and immune invasion. We subsequently analyzed cisplatin (Figure 5D) and paclitaxel (Figure 5E) IC50 scores in both groups and found that the low ACOT13 expression group was more likely to develop cisplatin resistance (*p* < 0.01). The TMB score of OSC patients was also analyzed, showing a positive correlation between ACOT13 expression and TMB score (Figure 5F, *p* < 0.001). For higher expression of ACOT13, the TMB score will be higher, suggesting that patients are more likely to benefit from immunotherapy.

### 3.5 Prediction of miRNA that regulate ACOT13 expression

In order to find miRNAs that may regulate ACOT13 expression, we analyzed the miRWalk database. Among the 15 miRNAs with targeted relationships, there was a significant negative correlation between hsa-miR-29b-1-5p and ACOT13 (*p* < 0.05). As shown in Table 2.

**TABLE 2:** The correlations between ACOT13 and miRNA.

Gene	miRNA	Cor	*p*-value
ACOT13	hsa-miR-125a-3p	−0.0658	0.204827
ACOT13	hsa-miR-330-5p	−0.06862	0.186067
ACOT13	hsa-miR-511-5p	−0.00347	0.946756
ACOT13	hsa-miR-625-3p	−0.05236	0.31318
ACOT13	hsa-miR-937-5p	0.047421	0.36109
ACOT13	hsa-miR-3187-5p	0.013056	0.801562
ACOT13	hsa-miR-4804-3p	0.115806	0.025312
ACOT13	hsa-miR-6821-3p	−0.02005	0.699571
ACOT13	hsa-miR-6885-5p	0.015572	0.764359
ACOT13	hsa-miR-6894-5p	0.042334	0.414941
ACOT13	hsa-miR-29b-1-5p	−0.13124	0.011179
ACOT13	hsa-miR-328-5p	0.01148	0.825102
ACOT13	hsa-miR-770-5p	0.082764	0.11053
ACOT13	hsa-miR-3198	0.097089	0.061037
ACOT13	hsa-miR-5003-3p	0.01829	0.724781

## 4 Discussion

Abnormal fatty acid metabolism in tumor microenvironment can affect tumor prognosis. Cao et al. (2022) used a prediction model constructed by 10 fatty acid metabolism-related genes to divide patients into high-risk and low-risk groups, and found that it could predict the overall survival and immunotherapy effect of ovarian cancer patients. However, the underlying mechanism of fatty acid metabolism on ovarian cancer progression has not been elucidated. It is necessary to study the specific molecules involved in the metabolism of fatty acids in more detail. ACOTs are a family of enzymes that exert various effects on cellular processes, but are primarily thought to contribute to lipid metabolism (Tillander et al., 2017). Fatty acids are degraded in mitochondria and peroxisome to provide energy for cell physiological activities in a process called β-oxidation. ACOTs is one of the key enzymes in the β-oxidation system, which can remove the short chain products that are unfavorable to the organism (Hunt and Alexson, 2008; Cooper et al., 2015). The imbalance of lipid metabolism also exerts a significant effect on the development of tumors. The high expression of ACOT1 was found in gastric cancer tissues, and the overall survival rate of patients with high expression of ACOT1 was greatly reduced, which may be realized by promoting the high expression of GLI family zinc finger 3 (GLI3) (Wang et al., 2018). In hepatocellular carcinomas, ACOT8 mRNA expression and gene copy number were increased, and the growth of cancer cells was inhibited when ACOT8 was knockdown (Hung et al., 2014). ACOT12 regulated the level of acetyl-CoA and histone acetylation in hepatocellular carcinoma cells. ACOT12 was underexpressed in hepatocellular carcinoma, and downregulation of ACOT12 can promote the metastasis of hepatocellular carcinoma by epigenetic induction of TWIST2 expression (Lu et al., 2019). ACOT11 and ACOT13 were highly expressed in lung adenocarcinoma and were related to poor prognosis (Hung et al., 2017). To our knowledge, this research firstly investigates ACOT13 expression and its prognostic value in ovarian cancer.

At present, the accepted standard treatment for epithelial ovarian cancer is surgery, integrated with platinum-based chemotherapy. About half of patients who receive a standard regimen of comprehensive staging surgery or satisfactory treatment of tumor cell reduction and a regular, adequate course of chemotherapy after surgery achieve a clinical complete response. However, even after the first standard treatment, 80% of patients with advanced cancer will relapse within 5 years due to symptoms, physical examination, imaging examination, or serological detection, which cannot detect tumor signs (Rochon et al., 2014; Korkmaz et al., 2016).

Therefore, it is necessary to identify more prognostic factors of ovarian cancer. This research firstly shows the high expression of ACOT13 in OSC and correlated with tumor staging. And patients with low ACOT13 expression were associated with a worse prognosis. Comprehensive discussion on the clinical characteristics of patients displayed that ACOT13 was an independent prognostic factor for OSC. We further researched the possible mechanism of ACOT13 affecting the prognosis of patients and discovered a significant positive correlation between ACOT13 and the immune checkpoint SIGLEC15. In recent years, immunotherapies such as immune checkpoint inhibitors have developed rapidly and have been investigated for the maintenance treatment of ovarian cancer, including anti-PD-1/PD-L1 and anti-CTLA-4 (Odunsi, 2017; Yang et al., 2020). SIGLEC15 was expressed on tumor-associated macrophages and had a similar domain composition to PD-L1. It was induced by macrophagocyte colony stimulating factor (M-CSF) (Takamiya et al., 2013). In pancreatic ductal adenocarcinoma, SIGLEC15 has been identified as a tumor-associated macrophage-associated immune checkpoint that polarizes M2-type macrophages and promotes tumor growth (Li et al., 2022). The high expression of ACOT13 in this study also suggested increased infiltration of macrophages. Notably, although SIGLEC15 shared many structural similarities with PD-L1, the expression of SIGLEC15 (which was inhibited by interferon-γ) was negatively correlated with the expression of PD-L1 (which was induced) (Wang et al., 2019). This may provide another idea for patients who do not react well to treatment with PD-1/PD-L1 inhibitors. Nevertheless, the specific mechanism of SIGLEC15 in ovarian cancer requires further study. Paclitaxel and platinum-based chemotherapy drugs are crucial in treating of ovarian cancer. We found that patients with low expression of ACOT13 had a high cisplatin IC50 score, which may be one of the reasons for the poor prognosis and requires our attention in the treatment. TMB is related to the generation of neoantigens triggering antitumor immunity and can be used to choose patients benefited by immune checkpoint inhibitor therapy (Gubin et al., 2015; Allgäuer et al., 2018). In this study, ACOT13 was positively correlated with TMB, and patients with low expression of ACOT13 may have poor efficacy of immune checkpoint inhibition. miRNAs can control gene expression at the post-transcriptional level. We observed that among the miRNAs that have a targeting relationship with ACOT13 mRNA, only hsa-miR-29b-1-5p has a significant negative correlation, which may regulate the expression of ACOT13 and further affect the prognosis of patients.

In fact, there are some limitations that need to be mentioned based on the current research. First, additional *in vivo* and *in vitro* studies are required to clarify the mechanism of ACOT13 in the occurrence of ovarian cancer, and this is where we will work in the future. Secondly, although the established nomogram has a certain predictive effect, C-index is not impressive enough. Prediction of tumor prognosis is difficult and requires multiple biomarkers to act together, but this does not affect the value of ACOT13 in ovarian cancer prognosis.

## 5 Conclusion

In summary, this study comprehensively analyzed the correlation of ACOT13 expression, prognosis, immune checkpoint, first-line chemotherapy IC50 score, and TMB in OSC. The results indicate that low expression of ACOT13 has a poor OS, PFS and DSS in OSC, and ACOT13 is an independent prognostic factor in OSC. In the future, further research is required on the potential mechanism of ACOT13 in the pathogenesis of OSC and its clinical application. For additional requirements for specific article types and further information please refer to “Article types” on every Frontiers journal page.

## Data Availability

Publicly available datasets were analyzed in this study. This data can be found here: https://portal.gdc.cancer.gov; https://commonfund.nih.gov/gtex; https://www.proteinatlas.org/ENSG00000112304-ACOT13/tissue/ovary; https://www.proteinatlas.org/ENSG00000112304-ACOT13/pathology/ovarian+cancer#ihc.
