# Supplementary material for: Low expression of acyl-CoA thioesterase 13 is associated with poor prognosis in ovarian serous cystadenocarcinoma
Source: Front Genet. 2023 Jun 22;14:1213022. doi: 10.3389/fgene.2023.1213022 (PMC10323136; doi:10.3389/fgene.2023.1213022)
Supplement: Supplementary file 1 [file DataSheet1.docx]

Supplementary Material

Low expression of acyl-CoA thioesterase 13 is associated with poor prognosis in ovarian serous cystadenocarcinoma

Xiaofeng Lv, Weijiao Wang Wan Xie, Silu Meng, Xiaoyu Liu, Yuhuan Liu, Lili Guo, Changyu Wang

*** Correspondence:** Corresponding Author: Changyu Wang Email: [tjwcy66@163.com](mailto:tjwcy66@163.com)

## Supplementary Figures


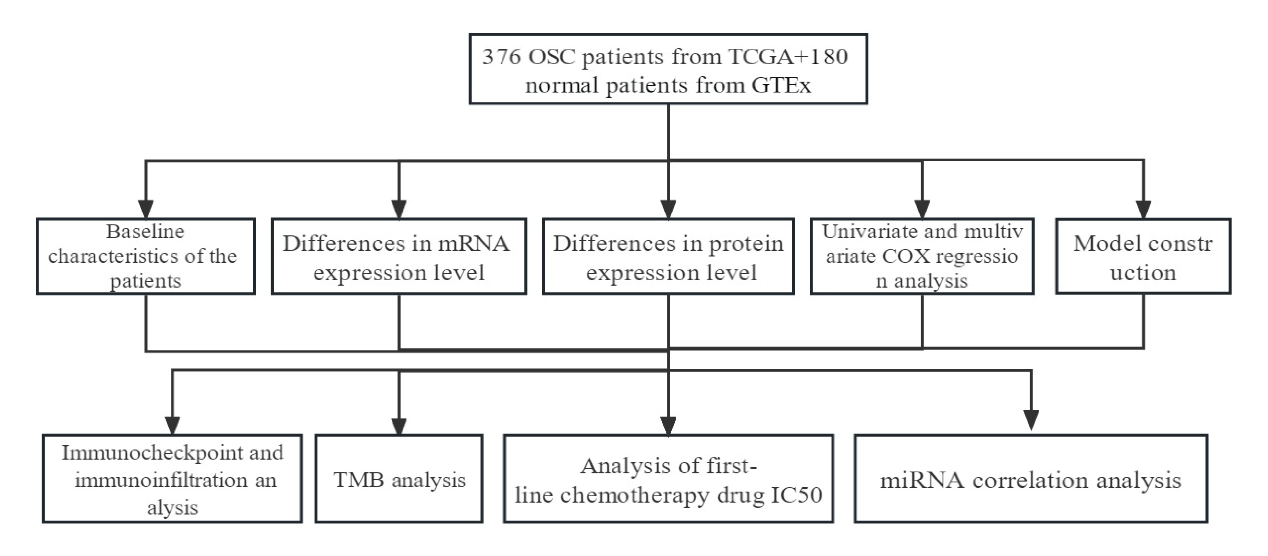


**Supplementary Figure 1.** Flow diagram of this study.


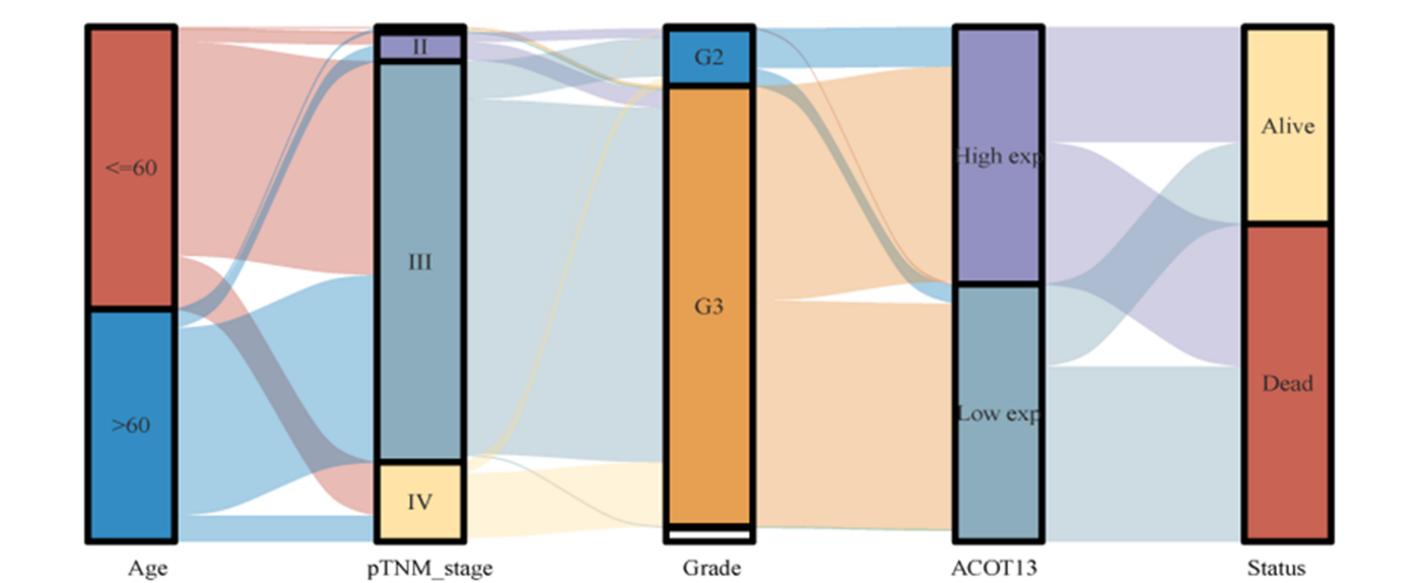


**Supplementary Figure 2.** The sankey diagram of ACOT13 and clinical characteristics. Each row represents a feature variable, different color represents different typing or stage, lines represent the distribution of the same sample in different feature variables.


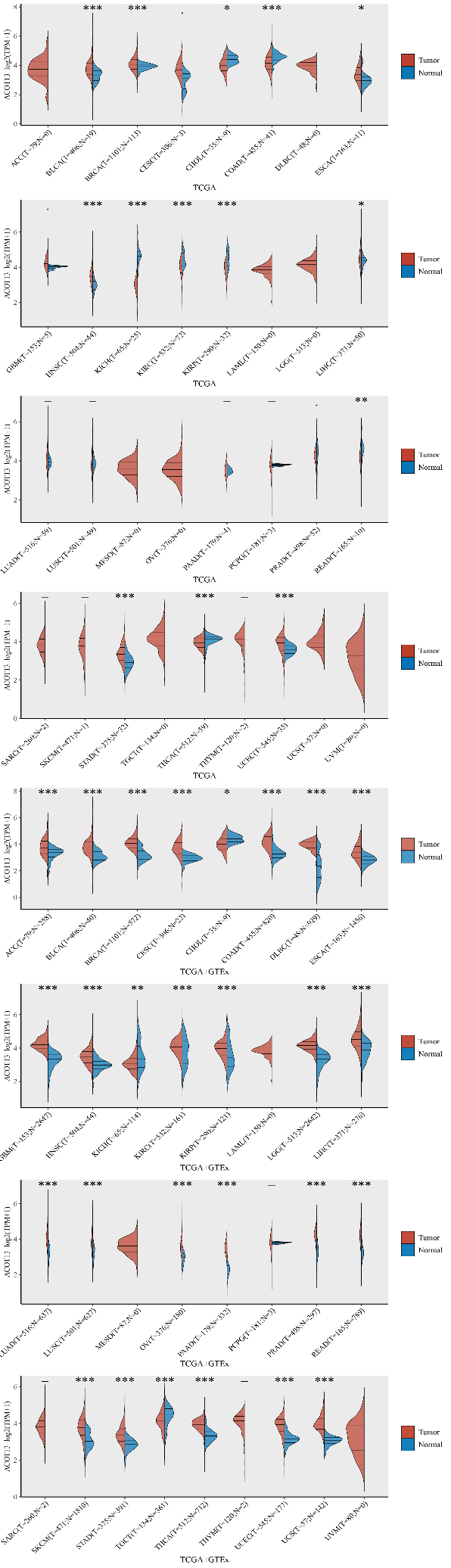


**Supplementary Figure 3.** Differential expression of ACOT13 in pan-carcinoma.


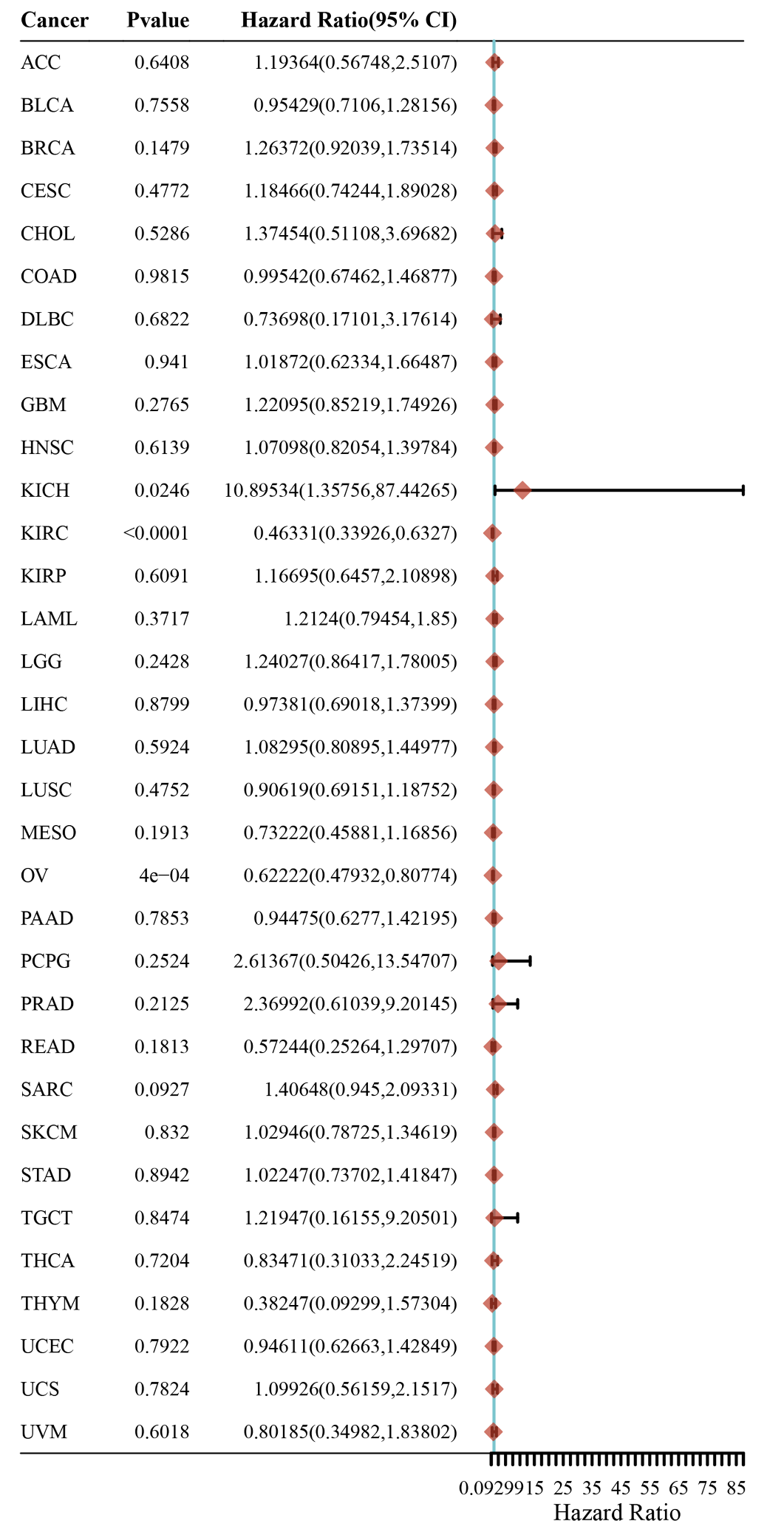


**Supplementary Figure 4.** Univariate cox regression analysis of ACOT13 in pan-carcinoma.
